# Conflict and COVID-19: a double burden for Afghanistan’s healthcare system

**DOI:** 10.1186/s13031-020-00312-x

**Published:** 2020-09-22

**Authors:** Don Eliseo Lucero-Prisno, Mohammad Yasir Essar, Attaullah Ahmadi, Xu Lin, Yusuff Adebayo Adebisi

**Affiliations:** 1 Department of Global Health and Development, London School of Hygiene and Tropical Medicine, London, UK; 2 Faculty of Management and Development Studies, University of the Philippines (Open University), Los Banos, Laguna Philippines; 3 Kabul University of Medical Sciences, Kabul, Afghanistan; 4 Department of Thoracic Surgery, the First Affiliated Hospital, Zhejiang University School of Medicine, Hangzhou, Zhejiang Province People’s Republic of China; 5 Faculty of Pharmacy, University of Ibadan, Ibadan, Nigeria

## Abstract

The novel coronavirus disease (COVID-19) has put an additional strain on Afghanistan’s weak healthcare system. Prior to the pandemic, the government and its allies had already problems in providing high quality health services for the people in Afghanistan because of inadequate facilities, insecurities, and ongoing conflicts. This year, COVID-19 exacerbated the situation and overwhelmed the healthcare system even further. As predicted, an influx of migrants suspected of having COVID-19 contributed to community transmission and led to an increase of cases across the country. A series of deadly attacks on civilians and healthcare workers in the country poses an additional burden, and severely weakens healthcare structures in times where health services are indispensable. These circumstances make evident that the international community needs to provide more support for Afghanistan’s healthcare system and pass the United Nations resolution for a ceasefire in the country.

## Background

COVID-19 is a global public health emergency [1]. The COVID-19 epidemic started in Afghanistan on 24 February 2020 in Herat. Cases of COVID-19 increased in the community after economic migrants started to return back to Afghanistan from Iran, a country badly hit by COVID-19 [2]. According to the International Organization for Migration, more than 400,000 migrant workers returned to Afghanistan from Iran and Pakistan [3]. As of 13 August 2020, there were 37,424 confirmed cases, with 1363 deaths across the 34 provinces of the country. The true numbers are likely to be higher than the official ones due to insufficient resources and testing capacity (97,778 samples have been tested as of 13 August 2020) [4] as well as lack of a national death register. Kabul, Herat, Balkh, Kandahar, and Nangarhar had reported the highest number of cases. Modelling shows that the cases will spike over the coming weeks as the peak has not yet been reached [5]. This article aims to provide a commentary on COVID-19 and conflict in Afghanistan.

## Commentary

In Afghanistan, limited COVID-19 testing laboratory capacity remains a challenge and hampers containment efforts. There are currently 11 COVID-19 testing laboratories—five in Kabul (National Public Health Lab, National Veterinary Lab, Afghan-Japan Hospital, and Military Hospital and FMIC), and one each in Herat, Kandahar, Nangarhar, Balkh, Paktya, and Kunduz [6]. This is insufficient to understand the extent of community spread, and the size of the pandemic in the country. In preparation of a possible increase of patients needing hospital treatment, Afghanistan is making efforts to increase its healthcare capacity as well. In Herat, a 100-bed COVID-19 hospital was opened in the early days of the pandemic. University dormitories and the Darulaman Palace with 300 beds were converted into isolation centres [6]. However, this won’t be sufficient and more needs to be done for an effective response.

Afghanistan’s healthcare system is ill-prepared to contain the virus and to deal with a high number of patients needing care for COVID-19. Healthcare professionals are lacking. According to WHO, there are 9.4 health professionals for every 10,000 people of which 1.9 are doctors only [2]. Healthcare workers in the country pay a high price for battling the pandemic with inadequate equipment and facilities. In Kunduz Regional Hospital in north-eastern Afghanistan, about 70 doctors and nurses were reported to be infected with COVID-19 [7] because of lack of personal protective equipment. As of 13 August 2020, infected healthcare workers comprised almost 10% of the total number of confirmed cases in Afghanistan [4]. Some regular routine appointments including vaccinations of preventable diseases such as polio were cancelled due to COVID-19 restrictions, resulting in surges [8].

Over the years, Afghanistan has experienced violence and conflict. A 2018 report shows that more than 10,000 civilians exposed to violence and conflict were either injured or killed, and over 365,000 people were displaced from their homes [9]. The same report stated that 140 health facilities serving two million Afghans were forcedly closed by armed groups. During the COVID-19 pandemic, there were a number of bombings in Afghanistan, further hampering containment of COVID-19. Conflict in the southern part of the country displaced around 10,000 people this year and the Humanitarian Access Group reports that a total of 227 incidents impeding access of humanitarians have occurred as well [5]. In Nangarhar province, 24 were killed and many were injured by a suicide bomber during a funeral [10]. This came a day after four civilians including a child were wounded when roadside bombs exploded in the northern district of Kabul. In March, a Sikh temple in the capital was attacked by a gunman and 25 people died [10].

On 12 May 2020 in Dasht-e-Barchi in southwest Kabul, Afghanistan, a 55-bed government-run maternity hospital was attacked claiming the lives of 24 mothers, babies, and healthcare professionals [11, 12]. This outrageous attack was planned on Médecins Sans Frontières (Doctors Without Borders) which were assisting the hospital in providing healthcare. This happened while the COVID-19 pandemic burdened an already fragile healthcare system that tries to control the spread of the virus, and cope with an increased number of patients requiring access to health services. In a country beset with major protracted wars and increased political and economic instability for decades to come, bombing of healthcare facilities, killing health personnel and patients during the time of a pandemic is the last thing that a country desires. This incident, eventually, forced Doctors Without Borders (MSF) to leave the hospital, depriving the needs of many mothers and their babies.

## Conclusion

The continued war and conflict amidst the COVID-19 pandemic is inducing a gruesome scenario for the people of Afghanistan. The current statistics point to further surges of COVID-19 cases in the future. Along with COVID-19, the number of wounded citizens also imposes further pressure on the weak healthcare systems. The ongoing crisis highlights the need to the global community to further work together to support Afghanistan’s healthcare system and pass the United Nations resolution for a ceasefire in the country.

## Data Availability

Not applicable.

## Abbreviations

*COVID-19*: Novel coronavirus disease
UN: United Nations
